# BH3-Only protein bmf is required for the maintenance of glucose homeostasis in an *in vivo* model of HNF1*α*-MODY diabetes

**DOI:** 10.1038/cddiscovery.2015.41

**Published:** 2015-10-05

**Authors:** S Pfeiffer, L Halang, H Düssmann, MM Byrne, JHM Prehn

**Affiliations:** 1 Department of Physiology and Medical Physics, Centre for Systems Medicine, Royal College of Surgeons in Ireland, 123 St. Stephen’s Green, Dublin 2, Ireland; 2 Department of Endocrinology, Mater Misericordiae University Hospital, 30 Eccles Street, Dublin 7, Ireland

## Abstract

Heterozygous loss-of-function mutations in the hepatocyte nuclear factor 1*α* (HNF-1*α*) gene can lead to diminished amounts of functional HNF-1*α*, resulting in the onset of a particularly severe form of maturity-onset diabetes of the young (MODY). We have previously shown that induction of a dominant-negative mutant of HNF-1*α* (DNHNF-1*α*) results in the activation of the bioenergetic stress sensor AMP-activated protein kinase (AMPK), preceding the onset of apoptosis and the induction of pro-apoptotic Bcl-2 homology domain-3-only protein Bmf (Bcl-2-modifying factor) as a mediator of DNHNF-1*α*-induced apoptosis. Through the knockout of *bmf* in a transgenic mouse model with DNHNF-1*α* suppression of HNF-1*α* function in pancreatic beta-cells, this study aimed to examine the effect of loss-of-function of this BH3-only protein on the disease pathology and progression, and further elucidate the role of Bmf in mediating DNHNF-1*α*-induced beta-cell loss. Morphological analysis revealed an attenuation in beta-cell loss in *bmf*-deficient diabetic male mice and preserved insulin content. Surprisingly, *bmf* deficiency was found to exacerbate hyperglycemia in both diabetic male and hyperglycemic female mice, and ultimately resulted in a decreased glucose-stimulated insulin response, implicating a role for Bmf in glucose homeostasis regulation independent of an effect on beta-cell loss. Collectively, our data demonstrate that Bmf contributes to the decline in beta-cells in a mouse model of HNF1A-MODY but is also required for the maintenance of glucose homeostasis *in vivo*.

## Introduction

Maturity-onset diabetes of the young (MODY) is a heterogeneous group of monogenic, non-insulin-dependent diabetes mellitus, characterized by autosomal dominant transmission and the development of severe hyperglycemia generally before the age of 25 years.^1^ Of these, hepatocyte nuclear factor 1*α* (HNF1*α*) -MODY is the most common and most severe. Patients are characterized by severe progressive hyperglycemia and beta-cell dysfunction, with impaired glucose-stimulated insulin secretion response by pancreatic beta-cells in contrast to insulin resistance by target tissues displayed by most NIDDM patients.^1,2^

HNF-1*α* is a dimeric, homeodomain-containing transcription factor involved in the control of expression of a wide variety of tissue-specific genes in the kidney, liver, spleen, intestine and pancreas, such as glucose transporter (Glut2) and insulin in the pancreatic beta-cell.^3–7^ Mutations the HNF-1*α* protein can lead to diminished amounts of functional HNF-1*α* through either haploinsufficiency or a dominant-negative mechanism and onset of the HNF1*α*-MODY phenotype.^3,8,9^ Several *in vitro* and *in vivo* models of various forms of MODY have been established utilizing these mutations to further elucidate the molecular mechanisms and progressive beta-cell dysfunction attributed to excessive beta-cell apoptosis observed in HNF1*α*-MODY.^10–12^

Apoptotic cell death is a hallmark of pancreatic beta-cell loss observed in all forms of diabetes mellitus, leading to the development of clinically overt disease.^13,14^ We and others have implicated caspase-dependent apoptotic pathways, along with activation of pro-apoptotic Bcl-2 protein family members such as Bax, Bim, Bad and Puma as essential for the initiation of beta-cell apoptosis both *in vitro* and *in vivo*.^10,15–19^ Previously, we demonstrated induction of pro-apoptotic BH3-only protein Bmf (Bcl-2-modifying factor) expression during energetic stress in insulin-secreting cells and in the islets of dominant-negative HNF-1*α* (DNHNF-1*α*)-expressing transgenic mice, identifying Bmf as a primary mediator of DNHNF-1*α*-induced apoptosis.^15^ Bmf acts as an indirect activator of apoptosis, containing a single BH3 domain that interacts with prosurvival Bcl-2 proteins Bcl-2, Bcl-xL and Bcl-w, preventing sequestration of direct activators such as Bid, Bim and Puma^20,21^ and has been shown to be induced as a result of AMP-activated protein kinase (AMPK) activation in response to bioenergetic stress to stimulate apoptosis,^15,22^ as well as having roles in autophagy and in cell death induced by high glucose levels in *in vivo* models of diabetes.^23–25^ In addition, Bmf has been shown to be induced post-transcriptionally through enhanced translation under conditions that cause repression of the CAP-dependent translation machinery, such as hyperglycemia.^22,26^

The current study was undertaken to elucidate the role of Bmf in mediating the progressive HNF-1*α* mutation-induced beta-cell death associated with HNF1*α*-MODY. Utilizing an established transgenic mouse model of HNF1*α*-MODY with a specific beta-cell-targeted DNHNF-1*α* mutation shown to result in glucose intolerance and overt diabetes,^11,12^ *bmf* expression was knocked out to examine the effect of loss-of-function of this BH3-only protein on disease pathology and progression, and further elucidate the role of Bmf in mediating DNHNF-1*α*-induced apoptosis.

## Results

### Gene deficiency in *bmf* restores beta-cell mass and partially preserves insulin content in male DNHNF-1*α* transgenic mice

**Figure 1:**
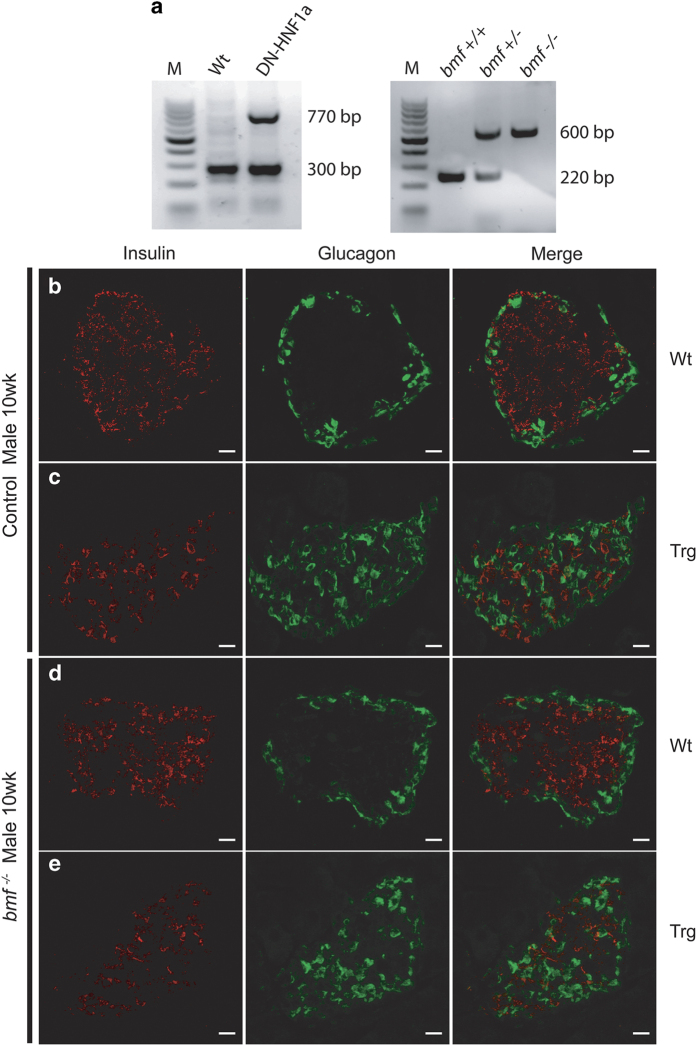
*bmf* deficiency partially rescues beta-cells in male DNHNF-1*α*-induced diabetic islets. (**a**) Representative standard PCR analysis of genomic DNA for DNHNF-1*α* and *bmf* genotyping illustrating predicted band size. M, 100-bp marker. (**b**–**e**) The effect of *bmf* knockout on pancreatic islet organization was assessed in 10-week-old male mice. Representative images of *bmf*-expressing (**b** and **c**) and *bmf*-deficient (**d** and **e**) islets stained with anti-insulin and anti-glucagon antibodies for the identification of alpha-cell (green) and beta-cell(red) localization and organization within the pancreatic islet. *n*=6 islets per pancreas from *n=*3 per group. Scale bar, 50 *μ*m.

In order to examine the role of Bmf in the progressive beta-cell dysfunction attributed to excessive beta-cell apoptosis observed in HNF1A-MODY, we introduced a *bmf* deficiency in a transgenic mouse model suppressed HNF-1*α* function in the pancreatic beta-cells^11,12^ to generate a transgenic *bmf-*deficient model of HNF1*α*-MODY (Figure 1a).

To assess the effect of *bmf* deletion on the pancreas, immunohistochemical analyses for insulin and glucagon expression were performed on pancreatic sections of *bmf-*expressing (DNHNF-1*α*^*bmf+/+*^) and *bmf*-deficient (DNHNF-1*α*^*bmf−/−*^) transgenic mice for analysis of islet structure. Representative images show double staining for 10-week-old male (Figures 1b–e) insulin- (red) and glucagon (green)-positive cells. In both sexes, deletion of *bmf* did not appear to have any effect on wild-type (WT) pancreatic sections that developed normally, displaying the typical islet architecture^27^ in both *bmf*-expressing (Figure 1b) and *bmf*-deficient (Figure 1d) WT groups. Upon examination of transgenic islets, disorganization of the islet is readily apparent, with a heterogeneous population of alpha-cells and beta-cells distributed throughout the islets in both DNHNF-1*α* groups (Figures 1c and e). Glucagon-positive alpha-cells can be seen to migrate into the center of the islet, with insulin-positive beta-cells no longer localized to the core but also evident on the periphery. Analysis of distribution of glucagon relative to insulin staining (quantified using distance mapping in ImageJ) confirmed no significant rescue in islet cell disruption in *bmf*-deficient transgenic islets (data not shown).

**Figure 2:**
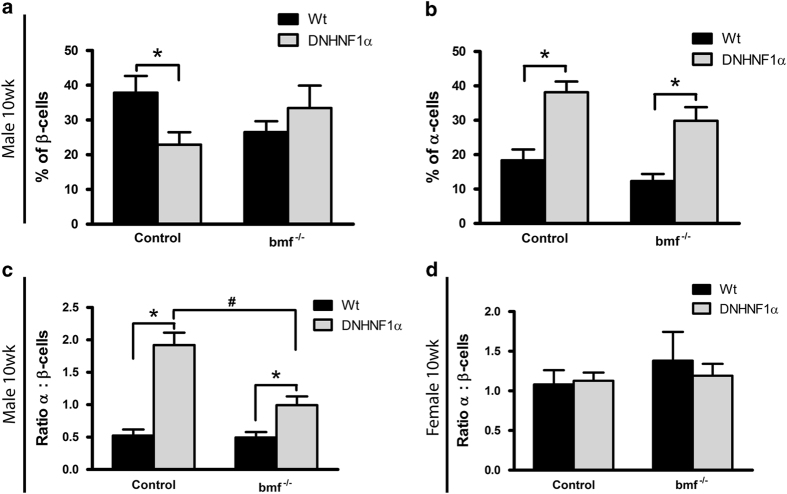
*bmf* knockout rescues beta-cell mass in male DNHNF-1*α* transgenic mice. Quantitative image analysis was used to assess the number of insulin-positive beta-cells (**a**) and glucagon-positive alpha-cells (**b**) in male 10-week-old immunostained pancreatic islets. (**c** and **d**), ratio of alpha-/beta-cells in male and female 10-week-old immunostained pancreatic islets, respectively. Data are presented as % of alpha- and beta-cells normalized to cell number. *n*=6 islets per pancreas from *n=*3 per group. *P<0.05 compared with litter-matched controls; ^#^*P*<0.05 compared with matched *bmf* -expressing controls (ANOVA, *post hoc* Tukey’s test).

Quantitative image analysis was used to assess the proportion of insulin- and glucagon-positive cells per islet. Whereas there were significantly fewer insulin-positive cells in male transgenic DNHNF-1*α*^*bmf+/+*^ (22.9±3.6%) compared with WT (37.8±4.8%) islets, deletion of *bmf* resulted in an increased number of insulin-positive cells in transgenic DNHNF-1*α*^*bmf−/−*^ islets (33.5±6.5%), comparable to that of WT controls (Figure 2a). Deletion of *bmf* had no appreciable effect on the increased glucagon-positive cell number in either colony (Figure 2b). These findings were reflected in the observed ratio of alpha/beta-cells; significant alpha-cell expansion and decreased beta-cell fraction in DNHNF-1*α*^*bmf+/+*^ islets were reflected in an increased alpha/beta-cell ratio (1.9±0.19) compared with WT^*bmf+/+*^(0.53±0.09), and the rescue in the number of insulin-positive beta-cells in DNHNF-1*α*^*bmf−/−*^ islets was reflected in a significantly decreased alpha/beta-cell ratio (0.99±0.13; Figure 2c). Similarly, we confirmed that no significant change in the ratio of alpha/beta-cells was observed in female islets in either group (Figure 2d), corresponding to the synchronously decreased alpha- and beta-cell fractions seen in female transgenic DNHNF-1*α*^*bmf−/−*^ islets.

**Figure 3:**
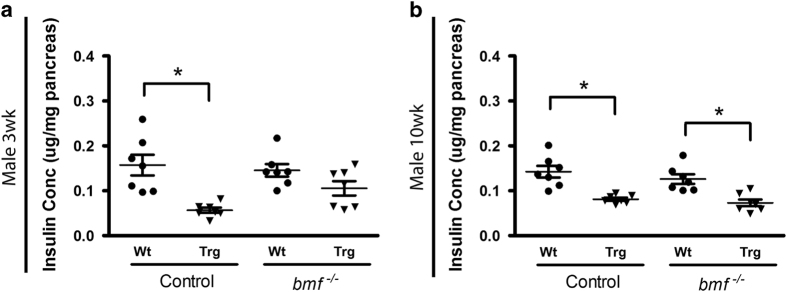
*bmf* knockout partially preserves pancreatic insulin content with rescue of beta-cell mass. Total pancreatic insulin content was measured in non-fasted male mice at (**a**) 3 and (**b**) 10 weeks of age by insulin ELISA. Data presented as mean±S.E.M. from *n*=7 per group. *P<0.05 compared with litter-matched controls (ANOVA, *post hoc* Tukey’s test).

In order to examine whether the increased cell mass observed was associated with increased pancreatic insulin content, we measured total pancreatic insulin content in non-fasted WT and DNHNF-1*α* mice aged 3 and 10 weeks. Pancreatic insulin content in *bmf*-expressing DNHNF-1*α* transgenic 3-week males was significantly lower (36% of WT^*bmf+/+*^control); however, insulin content of DNHNF-1*α*^*bmf−/−*^ transgenic mice was not significantly reduced (72.44% of WT^*bmf−/−*^ control, *P=*0.3), correlating with an increased beta-cell mass (Figure 3a). Subsequently, by 10 weeks rescue by deletion of *bmf* was no longer apparent, with insulin content significantly lower in both *bmf*-expressing (57.1% WT^*bmf+/+*^control) and *bmf*-deficient (57.9% of WT^*bmf−/−*^ control) DNHNF-1v transgenic groups (Figure 3b).

### Deletion of *bmf* worsens glycemic control in DNHNF-1*α* transgenic mice

**Figure 4:**
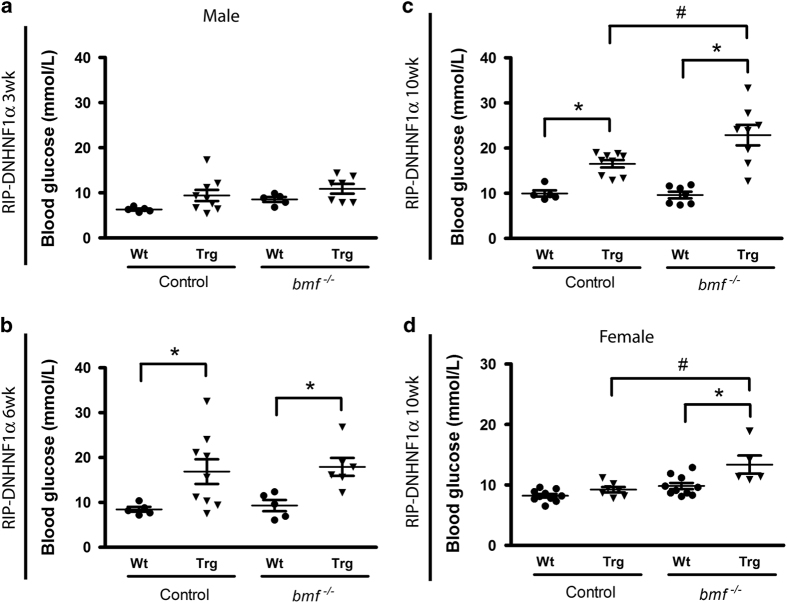
*bmf* knockout increases non-fasting blood glucose levels. Non-fasted blood glucose levels were measured at 3- (**a**), 6- (**b**) and 10-week-old (**c**) male and female (**d**) mice. Transgenic DNHNF-1*α* mice deficient in *bmf* demonstrated increased blood glucose at 10 weeks compared with matched *bmf*-expressing transgenic DNHNF-1*α* controls in both colonies. Data presented as mean±S.E.M. from *n*=5–10 per group. *P<0.05 compared with litter-matched controls; ^#^*P*<0.05 compared with matched *bmf*-expressing controls (ANOVA, *post hoc* Tukey’s test).

Non-fasted blood glucose levels were monitored over a 10-week period. While no difference was observed in 3-week males (Figure 4a), by 6 weeks a significantly increased blood glucose level was apparent in both DNHNF-1*α bmf*-expressing (16.9±2.8 mmol/l) and *bmf*-deficient (17.9±2.0 mmol/l) groups compared with WT controls (8.5±0.6 and 9.3±1.2 mmol/l, respectively); no distinguishable difference was observed between *bmf*-deficient and *bmf*-expressing control groups (Figure 4b). By 10 weeks, deletion of *bmf* significantly worsened already high blood glucose levels in DNHNF-1 ^*bmf−/−*^ mice (22.9±2.3 mmol/l) compared with DNHNF-1*α*^*bmf+/+*^ control group (16.5±0.8 mmol/l) despite the rescue in beta-cell mass (Figure 4c).

To examine whether decreased glycemic control was related to beta-cell loss, non-fasted blood glucose levels in female mice were also monitored. Interestingly, female DNHNF-1*α*^*bmf−/−*^ mice also displayed increased glucose levels (13.36±1.5 mmol/l) compared with DNHNF-1*α*^*bmf+/+*^ control group (9.23±0.44 mmol/l) at 10 weeks *versus* WT controls (9.8±0.5 and 8.2±0.3 mmol/l, respectively; Figure 4d).

**Figure 5:**
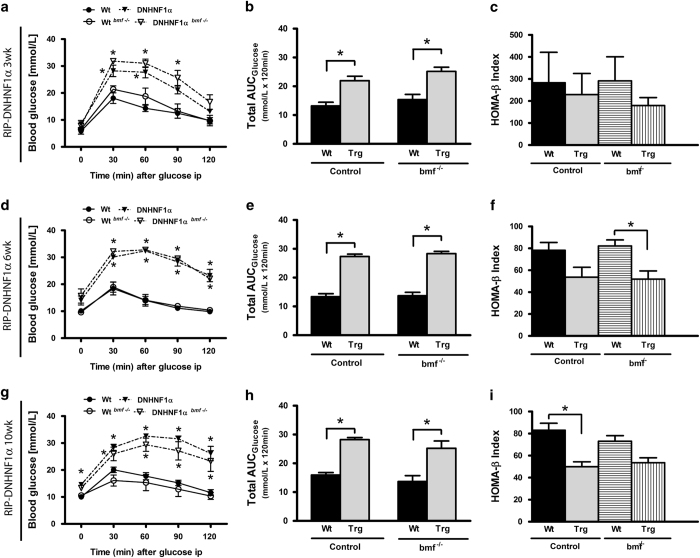
*bmf* deficiency does not attenuate increased serum glucose concentrations in male DNHNF-1*α* transgenic mice during intraperitoneal glucose tolerance test. Change in the blood glucose from fasting levels was determined over a 2-h period after i.p. injection of glucose in male 3- (**a**–**c**), 6- (**d**–**f**) and 10-week-old mice (**g** **–i**) fasted for 16 h. Blood glucose AUC (AUC_Glucose_) was used to evaluate the glucose clearance rate. Homeostatic model was used to assess beta-cell function (HOMA-*β*) during intraperitoneal glucose tolerance test. Data are presented as the mean change in blood glucose levels±S.E.M. *n=*6-7 per group. *P<0.05 compared with litter-matched controls (ANOVA, *post hoc* Tukey’s test).

Following these observations, in order to determine acute effects of *bmf* deficiency having an impact on insulin secretion and glucose tolerance, glucose tolerance tests were performed on 16-h fasted mice and changes in the blood glucose from basal fasting levels were measured over a period of 120 min. Both male DNHNF-1*α*^*bmf+/+*^ and DNHNF-1*α*^*bmf−/−*^ mice exhibited characteristic glucose intolerance from 3 weeks, with a 1.5-fold increased reactive serum glucose from 30 min (28.2±2.1 and 31.8±0.6 mmol/l, respectively) compared with WT controls (18.1±1.9 and 21.4±1.3 mmol/l; Figure 5a). This was illustrated with area under the curve (AUC) assessment of glucose response profiles, which were unaffected by deletion of *bmf* (Figures 5b and c). The reactive serum glucose profile was significantly increased at 6 weeks in both DNHNF-1*α*^*bmf+/+*^ and DNHNF-1*α*^*bmf−/−*^ groups, maintained at 120 min post administration (23.2±2.3 and 22.1±0.9 mmol/l, respectively) compared with WT controls (9.9±0.5 and 10.4±0.6 mmol/l; Figures 5d, e, f). By 10 weeks, both transgenic groups displayed increased basal (14.6±0.9 and 13.3±0.9 mmol/l) and reactive serum glucose (26.1±2.7 and 23.3±3.9 mmol/l, 120 min) compared with WT controls (10.0±0.4 and 10.6±0.7 mmol/l, 0 min; 11.6±1.1 and 10.4±1.3 mmol/l, 120 min; Figures 5g–i). Homeostatic model assessment of beta-cell function (HOMA-*β*) showed that, despite restoring decreased beta-cell mass, deletion of *bmf* had no significant effect on decreased basic insulin secretion function (*P=*1.0 between DNHNF-1*α*^*bmf+/+*^ and DNHNF-1*α*^*bmf−/−*^ groups at 3, 6 and 10 weeks).

Female mice displayed a similarly impaired glucose response profile; deletion of *bmf* worsened DNHNF-1*α* glycemic control at 3 weeks (17.7±2.7 mmol/l, 120 min) compared with DNHNF-1*α*^*bmf+/+*^ (10.7±2.2 mmol/l, 120 min) and similarly showed no significant effect on beta-cell function to 10 weeks.

### Attenuation of *bmf* expression decreases glucose-stimulated insulin secretion

**Figure 6:**
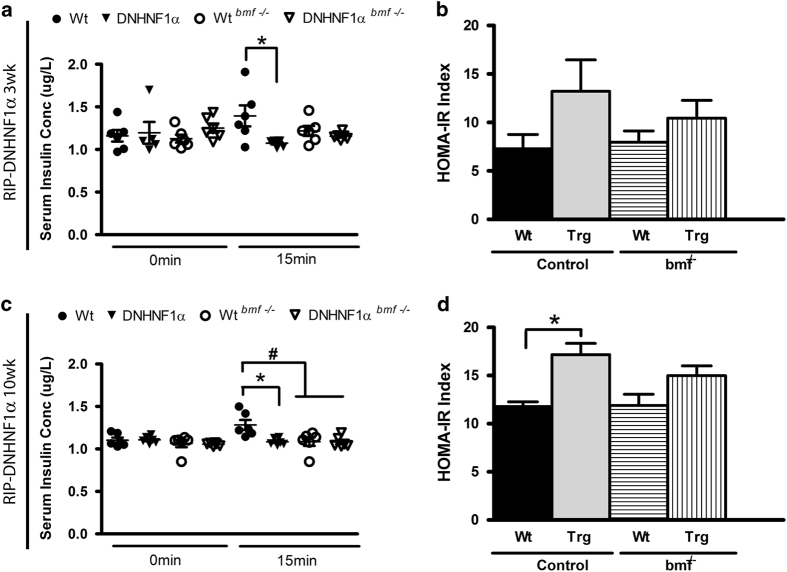
Deletion of *bmf* expression decreases serum glucose-stimulated insulin secretion. The change in serum insulin from fasting levels was determined at 0 and 15 min after i.p.GTT in male 3- (**a** and **b**) and 10-week-old mice (**c** and **d**) fasted for 16 h. Homeostasis model was used to assess insulin resistance (HOMA-IR) during intraperitoneal glucose tolerance test. Data are presented as the mean change in insulin levels±S.E.M. *n=*6-7 per group. *P<0.05 compared with litter-matched controls; ^#^*P*<0.05 compared with matched *bmf* -expressing controls (ANOVA, *post hoc* Tukey’s test).

Despite beta-cell preservation, the observed lack of corollary effect on glucose homeostasis led us to examine whether deletion of *bmf* in beta-cells leads to a specific insulin secretion defect. Basal and secreted insulin at 15 min was therefore also assessed in response to glucose challenge. Although no difference in fasted insulin levels was observed at 3 weeks, deletion of *bmf* did not confer any improvement in DNHNF-1*α*-decreased insulin response levels in DNHNF-1*α*^*bmf−/−*^ or WT^*bmf−/−*^ mice (*P=*1.0 compared with DNHNF-1*α*^*bmf+/+*^; Figures 6a and b). By 10 weeks, *bmf* deficiency remained ineffectual in having an impact on decreased insulin secretion observed in DNHNF-1*α*^*bmf+/+*^ (1.1±0.02 *μ*g/l) and DNHNF-1*α*^*bmf−/−*^ (1.1±0.03 *μ*g/l) and was also observed to decrease glucose-stimulated insulin secretion in WT^*bmf−/−*^ (1.1±0.04 *μ*g/l) compared with WT^*bmf+/+*^control (1.3±0.06 *μ*g/l; *P=*0.01 for all groups compared with WT*bmf+/+*; Figure 6c). Calculation of the HOMA index of degree of insulin resistance (HOMA-IR) demonstrated decreased insulin sensitivity in transgenic DNHNF-1*α*^*bmf+/+*^ mice (13.2±3.2) compared with WT^*bmf+/+*^ (7.3±1.5), not rescued by deletion of *bmf* (DNHNF-1*α*^*bmf−/−*^ *P=*1.0, WT^*bmf−/−*^ *P=*0.7).

## Discussion

Progressive beta-cell dysfunction and cell death, with impaired glucose-stimulated insulin secretion response and resulting hyperglycemia, is the hallmark and primary cause of diabetes and chronic related complications observed in HNF1*α*-MODY patients. Previous findings from our laboratory linking potent pro-apoptotic activity of Bmf to DNHNF-1*α*-induced apoptosis present it as an attractive target for investigation into NIDDM-associated stress-induced beta-cell death. Among pro-apoptotic BH3-only proteins implicated in cell death, the role of Bmf, induced as a result of AMPK activation in response to bioenergetic stress *in vitro*^15^ and mitochondrial ROS-mediated high glucose-induced upregulation in *in vivo* models of diabetes,^25^ still remains poorly investigated. Of note, Bmf has also been shown to have a role in endocrine tissue homeostasis of gastrointestinal epithelial cells.^28^ To investigate the consequences of *bmf* deletion on impaired HNF-1*α* function, we introduced a *bmf* deficiency in an established model of suppressed HNF-1*α* function in the beta-cells of mice. Our data conclude that deletion of Bmf results in a rescue of the progressive beta-cell death observed in HNF1*α*-MODY. However, attenuation of *bmf* expression results in an observable insulin-secretory defect, indicating that Bmf is required for insulin secretion, counteracting any rescues in beta-cell decrease.

Maintenance of islet architecture is important to the normal and pathological functioning of the islet; disruption to the preferential homologous contact between beta-cells carries functional implications for paracrine function, critically having an impact on beta-cell mass and insulin secretion.^29,30^ Interestingly, *bmf* deficiency resulted in a restoration of beta-cell mass in diabetic transgenic DNHNF-1*α* islets, which is reflected in a significantly decreased alpha/beta-cell ratio compared with that observed in *bmf*-expressing DNHNF-1*α* counterparts. Despite this partial recovery, the effect observed on increased glucose levels or insulin secretion response was negatory and the adjuvant heterologous disorganization of islet structure remained unaffected by attenuation of Bmf expression. Changes in the cellular location, resulting in disruption to specific interactions and connections with the extracellular matrix (ECM) and cell–cell junctions associated with tissue architecture, can alter signaling pathways associated with cell survival and result in anoikis through activation of BH3-only proteins and initiation of apoptosis to maintain tissue integrity and homeostasis.^31^ Previous studies have identified Bmf as a central regulator of anoikis,^20^ and significant upregulation of *bmf* has been observed upon anoikis induction through the disruption of cell–cell and cell–ECM contacts in human intestinal epithelial cells.^21^ Therefore, it is possible that Bmf may directly regulate anoikis in response to loss of, or inappropriate, cell–cell or cell–ECM interactions by acting as a sensor for actin cytoskeleton integrity in addition to its role as a mediator of energetic stress-induced apoptosis.

Previous studies aiming to elucidate the roles of BH3-only proteins as key modulators of beta-cell apoptosis have implicated members such as Bim and Puma in beta-cell apoptosis but also demonstrated an extensive degree of reciprocal functional redundancy. Studies in mouse models of Pdx1 haploinsufficiency found increased expression of Bim and Puma, and suppression of these genes showed improved glucose tolerance, enhanced beta-cell mass and reduced apoptotic cell death both in *in vivo* and *in vitro.*^32^ Similarly, significantly higher levels of Bim and Puma mRNAs have been observed in islets of human donors with type 2 diabetes, and mice deficient in Bim and Puma were significantly protected from high glucose-induced islet cell death.^18,19^ Loss of other BH3-only proteins Bid or Noxa had no impact on glucose-induced apoptosis.^18,33^ It is therefore possible that other BH3-only proteins also contribute to beta-cell apoptosis in the DNHNF1*α* mouse model, in particular at late disease stages.

Examination of homeostatic non-fasted blood glucose found that deletion of *bmf* significantly aggravates DNHNF‐1*α*-induced hyperglycemia. This dysregulation was accompanied by declining pancreatic insulin content; however, it should be noted that the significant increases in blood glucose in *bmf*-deficient transgenic mice were not reflected in further reduced insulin content. In fact, at 3 weeks total pancreatic insulin content was initially preserved by deletion of *bmf*; however, this was no longer apparent by 10 weeks, most likely because of a compensatory mechanism whereby other BH3-only proteins substitute for Bmf deficiency as discussed above. Subsequent studies examining glucose tolerance demonstrated severe glucose intolerance in DNHNF‐1*α* transgenic mice, and *bmf* deficiency was not observed to have any restorative effect. Similarly, gene deficiency had no effect on the adherent glucose-stimulated insulin secretion response; despite the development of increased basal glucose levels and severe hyperglycemia during glucose challenge, *bmf* deficiency remained ineffectual in having an impact on decreased insulin secretion, even lowering the response of *bmf*-deficient WT mice.

The transcriptional upregulation of Bmf in response to energetic stress and high glucose levels *in vitro* and *in vivo* suggest a role for Bmf in the maintenance of glucose homeostasis mechanisms. Impaired glucose response profiles, combined with the failure of *bmf* deletion to confer any improvement in decreased insulin response levels in either DNHNF-1*α* or WT mice despite initially increased total pancreatic insulin content, implicate a 'day-time' role for Bmf in functional beta-cell insulin secretion unrelated to cell death. These overlapping functions of Bmf, in both glycemic control and beta-cell apoptosis, combine upon *bmf* deletion to present the preserved beta-cell mass with attendant hyperglycemic glucose-intolerant profiles that we observed. Other studies have demonstrated similar outcomes through manipulation of mediators of beta-cell apoptosis. Overexpression of anti-apoptotic Bcl-2 family protein Bcl-xl, despite preventing beta-cell apoptosis, resulted in consequentially impaired glucose-induced insulin secretion and hyperglycemia because of defective mitochondrial nutrient metabolism and insulin secretion signaling.^34^ Similarly, loss-of-function of Bcl-2 and Bcl-xL in single and double Bax–Bak knockout beta-cells was demonstrated to significantly augment glucose-induced insulin secretion and glucose-dependent metabolic signals, suggesting a dampening of beta-cell response to glucose by prosurvival Bcl-2 proteins and a role for core apoptotic proteins in beta-cell physiology.^35^ The mechanism responsible for the effects of Bmf on beta-cell function may be mediated though a defect in mitochondrial metabolism, which has a crucial role in the stimulus-secretion coupling of glucose-induced insulin secretion in pancreatic beta-cells. Given the subcellular localization of Bcl-xL to the mitochondrial membrane^36^ and effects on mitochondrial function,^37–40^ the loss of Bmf and its interaction with Bcl-xL, and indeed Bcl-2 and Bcl-w, may be related to disturbances in mitochondrial signaling-mediating glucose-induced insulin secretion.

Collectively, our data point to a role for Bmf in having an impact on pancreatic beta-cell survival, but also contributing to pancreatic beta-cell function independent of cell death signaling.

## Methods

### Gene-targeted mice

Beta-cell-specific suppression of HNF-1*α* function was achieved using the rat insulin promoter (RIP) II to directly drive targeted overexpression of a dominant-negative mutant of HNF-1*α* (DNHNF-1*α*) in transgenic mice, a generous gift from Professor C.Wollheim (Centre Médical Universitaire, Geneva, Switzerland).^3,11^ Targeted *bmf*^*−/−*^ mutant mice originally generated from C57BL/6-derived Bruce4 ES cells backcrossed onto a C57Bl/6 J background were provided by Professor Andreas Strasser (WEHI, Melbourne, Australia).^23^ To generate mice deficient for Bmf, *bmf*^*−/−*^ mice were crossed with RIP-DNHNF-1*α* mice to produce mice heterozygous for both alleles (RIP-DNHNF-1*α*^+/−^ *bmf*^+/−^) and were intercrossed to generate Bmf-deficient RIP-DNHNF-1*α*^+/−^ *bmf*^*−/−*^ mice. As controls, WT and heterozygous RIP-DNHNF-1*α* mice expressing endogenous *bmf* were used. All mouse strains were backcrossed for >10 generations on an inbred C57BL/6 background. Animal experiments were carried out under license from the Department of Health and Children (Ireland) and in accordance with the Principles of Laboratory Animal Care and local Research Ethics Committee.

### Genotype analysis

Wild-type, transgenic and knockout alleles for DNHNF-1*α* and *bmf* were confirmed using PCR analysis of genomic DNA extracted from tail snips using High Pure PCR Template Preparation Kit (Roche, Basel, Switzerland). Genotyping was performed using the following specific primers: 5′-GGAGTTCAGACTTCGCCGAGAG-3′, 5′-GGCTGGTCACAAAGTTTGACACTG-3′ (WT allele-specific); 5′-GGAGTTCAGACTTCGCCGAGAG-3′, 5′-GCAAGAGGCAAGCCCTTCACTTGG-3′ (mutant allele-specific) for *bmf* and 5′-CTGCTAACCATGTTCATGCCT-3′ (sense), 5′-TGAATTGCTGAGCCACCTCTC-3′ (mutant allele-specific reverse) for DNHNF-1*α*.

### Confocal microscopy and immunohistochemistry

Morphometric analysis was carried out on age- and sex-matched animals aged 10 weeks. Mice were killed by cervical dislocation and the pancreas was dissected out, snap-frozen and stored at −80 °C. Pancreatic cryostat sections (12 *μ*m) were processed for double-immunofluorescence staining for detection of insulin and glucagon. Briefly, sections were incubated 2 h with diluted polyclonal guinea pig anti-insulin (1 : 100, Dako Diagnostics, Glostrup, Denmark) or monoclonal rabbit anti-glucagon (1 : 100, Cell Signalling Technology, Danvers, MA, USA) antibodies at RT. Sections were subsequently incubated at RT for 1 h with an Alexa 568-conjugated anti-guinea pig or Alexa 488-conjugated anti-rabbit secondary antibody (1 : 500, Invitrogen, Waltham, MA, USA) and mounted in VECTASHIELD Mounting Media with DAPI (4′,6-diamidino-2-phenylindole; Vector Labs, CA, USA). Images were acquired on a Zeiss-LSM510 confocal microscope (Carl Zeiss, Jena, Germany) as previously described,^41^ and analysis was carried out using the ImageJ software (version 1.48, NIH, http://imagej.nih.gov/ij/). For analysis of pancreatic sections, z-stack images of each islet imaged were generated, and number of insulin- or glucagon-positive cells per islet and alpha/beta-cell ratio were determined using integrated z-projection. Percentage of insulin-positive or glucagon-positive cells within each islet was quantified by number of cells stained positive for the protein of interest normalized to cell number, and from this the ratio of alpha/beta-cells was calculated. Otsu was used to segment immunostained and background areas from another, and areas above threshold were then used for quantification. The cell number was determined by number of nuclei per islet.

### Pancreatic insulin content

Pancreatic insulin content was determined from 3- and 10-week age- and sex-matched animals. Snap-frozen pancreata were weighed and insulin was extracted with cold acid-ethanol. Briefly, pancreata were incubated O/N in acid-EtOH (1.5% HCl in 70% ethanol) at −20 °C, and then homogenized and incubated O/N at −20 °C. Samples were centrifuged 15 min 2000 r.p.m. at 4 °C and supernatant removed and neutralized with 1 : 1 volume TRIS (1M pH7.5). Insulin content in acid-ethanol supernatant was determined with Ultrasensitive Mouse Insulin ELISA (Mercodia AB, Uppsala, Sweden).

### Non-fasting glycemic blood measurements

Homeostatic blood glucose levels were measured in age- and sex-matched animals at 3-, 6- and 10-week time points using Bayer CONTOUR Blood Glucose Meter (Bayer Diabetes Care, Dublin, Ireland). A small drop of blood (~0.6 *µ*l) from the tail vein was collected and blood glucose value recorded (mmol/l). All procedures were carried out in a blinded manner.

### Intraperitoneal glucose tolerance tests

Age- and sex-matched mice aged 3, 6 and 10 weeks were fasted overnight for ~16 h, weighed and subsequently injected intraperitoneally with glucose (2 g/kg body weight). For glucose tolerance tests, blood glucose levels were measured at 0, 30, 60, 90 and 120 min post injection from tail vein blood as described above. For insulin release studies, blood was collected at 0 and 15 min post injection. Serum insulin levels were measured with Ultrasensitive Mouse Insulin ELISA (Mercodia AB). All procedures were carried out in a blinded manner.

### Insulin release studies

For insulin release studies, measurement of serum insulin concentration during Intraperitoneal glucose tolerance test (ipGTT) was performed by blood collection at 0 and 15 min post injection using a Microvette 200 Z-Gel (Sarstedt, Hildesheim, Germany), followed by centrifugation for serum separation and analysis. Samples were stored at −80 °C before measurement for insulin levels. Serum insulin levels were measured with Ultrasensitive Mouse Insulin ELISA (Mercodia AB).

### Statistical analysis

Statistics were carried out using the SPSS-IBM software (IBM, Armonk, NY, USA). Data are typically presented as mean±S.E.M., and one-way ANOVA followed by Tukey’s *post hoc* or nonparametric analyses (Kruskal–Wallis, Mann–Whitney *U*-test) were employed where appropriate to determine statistical significance. For ipGTT, the AUC was used to evaluate the blood glucose response profiles using the trapezoidal rule.^42^ Evaluation of beta-cell function (%) and degree of insulin resistance (IR) were calculated using the HOMA as follows: HOMA-*β*=(20 x fasting serum insulin (*μ*U/l)/fasting blood glucose (mmol/l)−3.5)% and HOMA-IR=(fasting serum insulin (*μ*U/l)xfasting blood glucose (mmol/l)/22.5), respectively.^43^

## Abbreviations

BH3: Bcl-2-homology domain-3
Bmf: Bcl-2-modifying factor
DNHNF1*α*: dominant-negative mutant of rat HNF1*α*
HNF1*α*: hepatocyte nuclear factor 1*α*
INS-1: rat insulinoma cells
ipGTT: intraperitoneal glucose tolerance test
NIDDM: non-insulin-dependent diabetes mellitus
RIP: rat insulin promoter
WT: wild type.
